# Ameliorative Potentials of Ginger (*Z. officinale* Roscoe) on Relative Organ Weights in Streptozotocin induced Diabetic Rats

**Published:** 2013-06

**Authors:** C. O. Eleazu, M. Iroaganachi, P. N. Okafor, I. I. Ijeh, K. C. Eleazu

**Affiliations:** 1 Department of Biochemistry, National Root Crops Research Institute, Umudike, Nigeria;; 2 Department of Food Science and Technology, Abia State Polytechnic, Aba, Nigeria;; 3 Department of Biochemistry, Michael Okpara University of Agriculture, Umudike, Nigeria

**Keywords:** diabetes, streptozotocin, rats, incorporated feeds, experimentation

## Abstract

The ameliorating potentials of ginger incorporated feed (10%) on the relative organ weights of Streptozotocin (STZ) induced diabetic rats was investigated. The experiment lasted for three weeks. Results show that administration of 10% ginger feed to the diabetic rats of group 3, resulted in a 29.81% decrease in their resulting hyperglycemia with a corresponding amelioration of elevated urinary protein, sugars, specific gravity as well as renal growth. In addition, administration of the ginger incorporated feeds to the diabetic rats of group 3, resulted in 9.88% increase in body weight with a corresponding 60.24% increase in growth compared with the non-diabetic rats administered standard rat pellets that had 6.21% increase in weight with a corresponding 60.14% increase in growth unlike the diabetic control rats that recorded 28.62% decrease in body weight with a corresponding 239.9% decrease in growth rates. Analysis of the chemical composition of the flour of the ginger incorporated feed indicated that it contained moderate amounts of moisture, crude fibre, alkaloids, saponins, tannins, Fe and Zn but considerable amounts of proteins, lipids, carbohydrates, ash, flavonoids, calcium, magnesium, potassium, phosphorous and energy value. There was no significant difference (*P*>0.05) in the liver and relative liver weights of the diabetic control rats and the diabetic -ginger treated rats. In addition, there were no significant differences in the kidney weights of the non-diabetic, diabetic control and diabetic treated rats (*P*>0.05) while there were significant differences in the relative kidney weights of the non-diabetic rats and the diabetic rats treated with ginger feeds (*P*<0.05). Results show that the use of ginger in the dietary management of diabetes mellitus could be a breakthrough in the search for novel plants that could prevent the development of diabetic glomerular hypertrophy.

## INTRODUCTION

Diabetes mellitus is one of the most challenging metabolic pandemic of the 21^st^ century that affects essential biochemical pathways in the body (carbohydrate, protein and lipid metabolism) (19) leading to the development of complications such as: renal disorders, neuropathy, retinopathy, ketoacidosis, and cardiovascular diseases. This disease arises either from a deficiency of pancreatic β-cells (as a result of an autoimmune disorder which destroys the beta cells of the pancreas to produce type 1) or insulin resistance which may be due to a number of defects in signal transduction ranging from abnormal insulin receptors to defects in glucose transporters (GLUT 4, which produces type 2) resulting in hyperglycemia (38).

During diabetes, the liver has been reported to decrease in weight due to enhanced catabolic processes such as glycogenolysis, lipolysis and proteolysis, which is the outcome of lack of insulin in the liver cells while the kidney has been reported to increase in weight due to glucose over-utilization and subsequent enhancement in glycogen synthesis (26), lipogenesis and protein synthesis. These changes may lead to serious microvascular renal complications, which involves a series of metabolic changes in the pathogenesis of diabetic nephropathy. In addition, diabetic glomerular hypertrophy constitutes an early event in the progression of glomerular pathology which occurs in the absence of mesengial expansion (22). Moreover, over 40% of diabetic patients world wide, develop severe diabetic nephropathy (4) and despite much research work, the diabetic kidney epidemic keeps increasing rapidly. Patients with diabetic kidney failure undergo either painful dialysis or kidney transplant (30), which is costly and harmful. On the other hand, the use of synthetic drugs in the management of the disease has been associated with serious side effects and researches towards the development of drugs with lesser side effects that could decrease the development of diabetic kidney damage has shown little success (21). This has therefore increased the search for medicinal plants that can arrest this crippling disorder.

The diets that are commonly used in the management of diabetes in Nigeria include: acha (*Digitaria exillis*), breadfruit (*Treculia africana*), beans (*Phaseolus vulgaris*) (9). However, diabetic patients have often complained of the monotony of staying on a particular diet (personal communication) and this has therefore increased the research into other plants with similar anti-diabetic potentials as the ones being used.

The ginger (*Zingiber officinale* Roscoe) rhizome is commonly found in most Nigerian homes where a small percentage of its flour (not more than 10%) is used mainly as a spice for flavoring a variety of dishes and drinks. Ginger has been identified as a herbal medicinal product with pharmacological effect. Ginger suppresses prostaglandin synthesis through inhibition of cyclooxygenase- 1 and cyclooxygenase- 2. Although it has been reported to be used in traditional medicine by other countries of the world in the management of diabetes (2), a greater percentage of the Nigerian populace are unaware of this. Moreover, there is paucity of information in literature on its use in the management of diabetic complications.

Since proper diet is critical in the management of diabetes mellitus and being that diabetic patients are eager and more willing to consume foods they are familiar with (16), we decided to commence a preliminary investigation on the ameliorating potentials of ginger on the altered relative organ weights of diabetic rats induced with Streptozotocin at a dosage of 65 mg/kg body weight.

## MATERIALS AND METHODS

### Plant materials

The ginger (*Zingiber officinale* Roscoe) variety (UGI) was freshly obtained at harvest from the National Root Crops Research Institute, Umudike, Nigeria. It was authenticated in the Department of Botany, Michael Okpara University of Agriculture, Umudike, Nigeria.

### Chemicals

Streptozotocin (STZ), DPPH (2,2-diphenyl-1-picrylhydrazyl) radical and standard quercetin were products of Sigma and Aldrich Chemical Company,UK. All other chemicals that were used in the experiment were bought from HosLab, Umuahia, Abia State, Nigeria and were of analytical grade.

### Processing of the plant materials

The samples were properly washed, peeled and oven dried at 50°C for 48 hours until constant weight was obtained before being pelletized and incorporated into the normal rat feeds.

### Proximate analysis

The moisture, crude protein, lipid, crude fibre and ash contents of the ginger incorporated feed were carried out using the methods of the Association of Analytical Chemists (3). Triplicate samples were incinerated in a muffle furnace (Thermodyn Type 1400 Furnace, Dubuoue, USA) at 600°C until a constant weight was obtained. Their carbohydrate contents were estimated by difference (3). The energy value of the test feed was calculated from the Atwater Formula of 4, 9 and 4 by multiplying the total carbohydrate content by 4, percentage lipid by 9, percentage protein by 4 respectively and taking the sum of the products.

### Phytochemical analysis

The gravimetric method of Harbone (14) was used in the determination of the percentage alkaloid contents of the ginger incorporated feed while the AOAC methods (3) were used in the determination of the flavonoid, saponin and tannin composition of the ginger incorporated feed.

### Mineral analysis

The atomic absorption spectrophotometer (Analyst 200, Perkin Elmer, Waltham, MA, USA) was used in the analysis of Fe, Zn, Mg and Ca, the flame photometric method was used for the analysis of K while the molybdate method (33) was used for the analysis of the phosphorous content of the ginger incorporated feed.

### Rapid Thin Layer Chromatrography (TLC) free radical scavenging screening

The TLC screening of the antioxidant activity of the methanolic extract of the ginger incorporated feed was determined using the DPPH method as proposed by Mensor *et al*. (25) with minor modifications. With the aid of a capillary tube, stock solutions (100 mg/ml) (instead of 1 mg/ml) of the extract were spotted on a silica gel Thin Layer Chromatographic (TLC) plate and developed with a solvent system of ethanol: methanol (90:10). After development, the chromatograms were dried and sprayed with a 0.3 mM solution of the stable DPPH free radical. The plates were visualized for the presence of yellow spots and the degree of activity was determined qualitatively from observation of the yellow colour intensity. Yellow spot formed (within 30 minutes of spraying) against a purple background was taken as a positive results. Quercetin was used as the positive control for this assay.

### Animal experiments

**Selection of animals.** Male albino rats of the Wistar strain (129.81-229.11 g) obtained from the animal house of the Department of Biochemistry, University of Nigeria, Nsukka, Enugu State, Nigeria, were used for the study. The animals were kept in metabolic cages in the animal house of the Department of Biochemistry, Michael Okpara University of Agriculture, Umudike, Nigeria. The rats were acclimatized for two weeks to their diets and water prior to the commencement of the experiment and were maintained under a constant 12-h light and dark cycle and an environmental temperature of 27-30°C. The experimental procedures were approved by the Ethical committee of Michael Okpara University of Agriculture, Umudike, Nigeria. The National Institutes of Health Principles of Laboratory Animal Care (29) were observed.

**Induction of diabetes.** Freshly prepared solution of streptozotocin (0.1 g dissolved in 5 ml of freshly prepared sodium citrate buffer 0.1 M, pH 4.5) was injected intraperitoneally to the animals at a dosage of 65 mg/kg body weight at fasting state. Blood was collected from the tail vein and the blood glucose concentration was analyzed prior to the commencement of the dietary feeding using a blood glucose meter (Double G glucometer, USA) and subsequently, twice in a week, throughout the duration of the experiment. The STZ-treated rats with fasting blood glucose levels >200 mg/dl after three [3] days of induction of STZ, were considered to be diabetic. The severity of diabetes was checked in the 24 hour urine samples of the STZ-treated rats using Urine Glucose Detection Strips (Clinistix, Bayer Health Care, USA) and Urine Reagent Strips for urinalysis (qualitative and quantitative) tests for glucose, protein, ketone and bilirubin (CONDOR-TECHO URS-10, Condor Teco Medical Technology Co., Ltd, China). The specific gravity of the urine samples was determined with a urinometer. The animals were also observed for physical activity such as excessive thirst (polydypsia) and excessive hunger (polyphagia).

**Experimental procedure.** The experimental rats with stable diabetic condition were then divided into 2 sub-groups (groups 2 and 3) while the non-diabetic group formed the first group as follows:

Group 1. Normal rats administered standard rat pellets (Non-diabetic control);
Group 2. Diabetic control rats;
Group 3. Diabetic rats treated with ginger incorporated feeds.

Their diets and water were both administered *ad libitum* for 21days, after which the rats were anesthetized with chloroform and their liver and kidney were collected and weighed. The body weights and feed intakes of the rats were recorded on a daily basis, using an electronic weighing balance (Model Scout Pro, Ohaus Corporation, USA) and were calculated as:

$$\text{Percentage change in weight}\;=\;\frac{\text{Initial weight}\;-\;\text{Final weight}}{\text{Initial weight}}\;\times \;100$$

Feed intake=Feed administered-Residue

While the percentage growth rate of the animals was calculated as:

$$\text{Percentage growth rate}\;=\;\frac{\text{Final weight}\;-\;\text{Initial weight}}{\text{Experimental duration}}\;\times \;100$$

Similarly, the percentage change in fasting blood glucose of the animals was calculated as:

$$\text{Percentage change in fasting blood glucose}\;(\text{FBG})\;=\;\frac{\text{Initial FBG}\;-\;\text{Final FBG}}{\text{Initial FBG}}\;\times \;100$$

Finally, the relative tissue weights of the animals were calculated as:

$$\text{Relative liver weight}\;(\text{g}/100\text{g})\;=\;\frac{\text{Total liver weight}}{\text{Final body weight}}\;\times \;100$$

$$\text{Relative kidney weight}\;(\text{g}/100\text{g})\;=\;\frac{\text{Total kidney weight}}{\text{Final body weight}}\;\times \;100$$

### Statistical analysis

Data was subjected to analysis using the Statistical Package for Social Sciences (SPSS), version 15.0. Results were presented as the means ± standard deviations of triplicate experiments. One way analysis of variance (ANOVA) was used for comparison of the means. Differences between means were considered to be significant at P<0.05 using the Duncan Multiple Range Test.

## RESULTS

The administration of STZ at a dosage of 65 mg/kg body weight to the rats of groups 2 and 3, produced stable diabetic condition within 3 days in all the rats. Administration of the ginger incorporated feed to the diabetic rats of group 3 resulted in a 29.81% decrease in the resulting hyperglycemia compared with the normal control and diabetic control rats (Table 1).

**Table 1:** Fasting blood glucose of diabetic and non-diabetic rats (mg/dl)

	WeeK 0	Week 1	Week 2	Week 3	PC (%)

Group 1	70.67 ± 10.60	87.00 ± 7.55	92.00 ± 8.00	93.67 ± 8.50	-7.67 (increase)
Group 2	104.00 ± 13.28^b^	352.67 ± 150.88^b^	355.00 ± 150.95^b^	308.00 ± 54.88^b^	12.67 (decrease)
Group 3	87.00 ± 7.94^a^	312.00 ± 48.08^b^	168.50 ± 58.69^c^	219.50 ± 111.01^c^	29.81 (decrease)

Values are presented as means ± SD. n=6; PC, Percentage change in fasting blood glucose calculated from week 1;

a *P*>0.05 in comparison with diabetic control within the group;

b *P*<0.05 in comparison with normal control within the group;

c *P*<0.05 in comparison with diabetic control within the group.

The diabetic rats of groups 2 and 3 had varying levels of glucose and protein in their urine by the 1^st^ and 2^nd^ week of the experimentation (Table 2) which is an indication of the severity of the diabetic condition of the animals. However, by the last week of the experimentation, administration of the test diet to the diabetic rats of group 3, resulted in their excretion of trace/low amounts of glucose and proteins in their urine.

**Table 2:** Biochemical parameters in the urine of diabetic and non-diabetic rats

	Week 0	Week 1	Week 2	Week 3

Group 1	Glucose: -ve	-ve	-ve	-ve
	Protein: Nil	Trace	Trace	Trace
	SPGR: 1.015 to 1.02	1.02	1.02 to 1.025	1.02 to 1.025
Group 2	Glucose: -ve	Trace to 2+	Trace to 2+	2+
	Protein: Nil	100 mg/dl	100 to 300 mg/dl	100 to 300 mg/dl
	SPGR: 1.03	1.06 to 1.07	1.03 to 1.04	1.025 to 1.03
Group 3	Glucose: Nil	Trace to +	-ve to 2+	-ve to trace
	Protein: Trace	30 to 100 mg/dl	30 to 100 mg/dl	Nil to 30 mg/dl
	SRGR: 1.02 to 1.025	1.05 to 1.07	1.04 to 1.07	1.02 to 1.06

-ve, negative or absent; +, positive or present.

The specific gravity of the urine of the diabetic rats in groups 2 and 3 was elevated, ranging from 1.06 to 1.07 by the 1^st^ and 2^nd^ weeks of the experimentation (Table 2). However, by the last week of the experimentation, administration of the test diet to the diabetic rats of group 3, resulted in the amelioration of the elevated specific gravity of their urine.

The liver weights of the diabetic rats of groups 2 and 3 showed a significant decrease (*P*<0.05) compared with the non diabetic rats while there were no significant differences (*P*>0.05) in the kidney weights of the diabetic rats of groups 2, 3 and the non-diabetic rats (Table 3).

**Table 3:** Organ weights and relative organ weights of diabetic and non-diabetic rats

	Liver weight (g)	Kidney weight (g)	Relative liver weight (g/100 g)	Relative kidney weight (g/100 g)

Group 1 (Control)	5.83 ± 0.30	1.33 ± 0.33	2.72 ± 0.16	0.61 ± 0.11
Group 2	4.57 ± 0.32^b^	1.27 ± 0.15^e^	3.70 ± 0.4^b^	1.02 ± 0.06^b^
Group 3	4.95 ± 0.21^b^	1.30 ± 0.28^e^	3.52 ± 0.02^b^	0.92 ± 0.18^c^

Values are presented as means ± SD. n=6;

b *P*<0.05 in comparison with normal control within the groups (column);

c *P*<0.05 in comparison with diabetic control;

e *P*>0.05 in comparison with normal control within the group.

The relative liver weights of the diabetic control rats and the diabetic rats administered ginger incorporated diets were not significantly different from each other (*P*>0.05) but differed significantly from that of the non-diabetic rats (*P*<0.05) (Table 3).

The relative kidney weights of the non-diabetic, diabetic control and diabetic rats administered ginger incorporated feed differed significantly from each other (*P*<0.05) (Table 3).

The body weights of the diabetic control rats decreased by 28.62% while the diabetic rats administered ginger incorporated feed, recorded 9.88% gain in weight compared with the non-diabetic rats that recorded 6.21% gain in weight (Table 4).

**Table 4:** Body weights of non-diabetic and diabetic rats (g)

	Week 0	Week 1	Week 2	Week 3	PC (%)	PG (%)

Group 1	208.37 ± 20.74	203.47 ± 19146	205.30 ± 20.19	216.10 ± 21.86	-6.21 (increase)	60.14 (i/c)
Group 2	194.67 ± 9.40^a^	175.97 ± 5.35^a^	144.90 ± 10.57^a^	125.60 ± 20.69^b^	28.62 (decrease)	-239.9 (d/c)
Group 3	140.35 ± 10.54^a^	128.10 ± 7.35^a^	124.50 ± 1.56^a^	140.75 ± 6.58^a^ ^b^	-9.88 (increase)	60.24 (i/c)

Values are presented as means ± SD. n=6; PC, Percentage change in weight calculated from week 1; PG, Percentage Growth Rate; i/c, Increase; d/c, decrease.

a *P*<0.05 versus normal control within the column;

b *P*>0.05 vs diabetic control within the groups (column).

The diabetic control rats recorded 239.9% decrease in growth rate, the diabetic rats administered ginger incorporated feed, recorded 60.24% increase in growth rate compared with the non-diabetic rats that recorded 60.14% increase in growth rate (Table 4).

The feed intake of the diabetic control rats and the diabetic rats administered ginger incorporated feed, increased by the last week of the experimentation (Table 5). In addition, the feed intake of the diabetic control rats was not significantly different from that of the non-diabetic rats (*P*>0.05) by the last week of experimentation while the feed intake of the diabetic rats administered ginger incorporated feed, was significantly different from that of the non-diabetic rats by the last week of experimentation (*P*<0.05) (Table 5).

**Table 5:** Feed intake of rats (g/week)

	Week 0	Week 1	Week 2	Week 3

Group 1	112.50 ± 2.59	108.13 ± 6.91	117.73 ± 9.45	118.33 ± 7.36
Group 2	115.78 ± 1.41^c^	95.37 ± 2.44^e^	108.70 ± 3.20^b^	116.22 ± 2.88^b^
Group 3	86.05 ± 4.60^a^	85.15 ± 0.21^e^	65.15 ± 2.62^a^	86.85 ± 0.35^a^

Values are presented as means ± SD. n = 6;

a *P*<0.05 in comparison with diabetic control;

b *P*>0.05 in comparison with normal control within the groups (column);

c *P*<0.05 in comparison with normal control;

e *P*<0.05 in comparison with normal control within the groups (column).

The composition of the ginger incorporated feed comprised of 10% ginger flour, 35.22% soybean flour, 15.65% vitamin mixture, 7.8% salt, 15.67% banana flavor and 15.67% groundnut oil.

The proximate composition of the ginger incorporated feed indicated that it contained on the average, 3.48% moisture, 21.67% ash, 1.52% crude fibre, 21.48% lipid, 10.25% crude protein, 43.13% carbohydrate and 406.82Kcal/100g of energy (Table 6).

**Table 6:** Proximate composition of ginger incorporated feed (%)

Parameter	MC	Ash	CF	Lipid	Crude protein	Carbohydrate	Energy value (Kcal/100 g)

Ginger	3.48 ± 0.10	21.67 ± 0.05	1.52 ± 0.20	21.48 ± 0.00	10.25 ± 0.01	43.13 ± 0.06	406.82 ± 0.20

MC, Moisture content; CF, Crude fibre.

The thin layer chromatographic screening of the methanolic/ethanolic extracts of the ginger incorporated feed indicated that it possessed strong antioxidant activities compared with standard quercetin (Table 7).

**Table 7:** Free radical scavenging activities of the methanolic/ethanolic extracts of ginger incorporated feed using Rapid DPPH TLC screening

Plant	Antioxidant activity	Intensity of spots

Ginger	Strong	+++
Quercetin	Strong	+++

The degree of activity, determined qualitatively from the observation of the yellow colour intensity: strong (+++).

**Figure 1:**
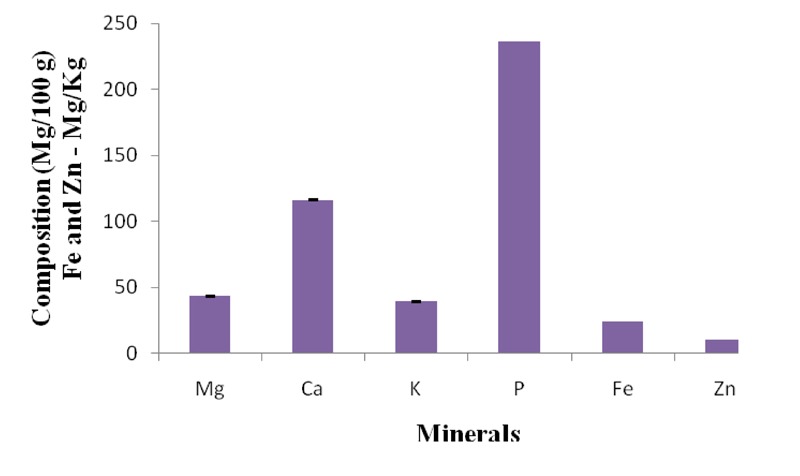
Mineral composition of ginger incorporated feed.

The mineral analysis of the ginger incorporated feed showed that it contained on the average, 43.55 mg/100g Mg, 116.38 mg/100g Ca, 39.47 mg/100g K, 236.40 mg/100g P, 2.47 mg/100g Fe and 1.08 mg/100g Zn (Figure 1).

**Figure 2:**
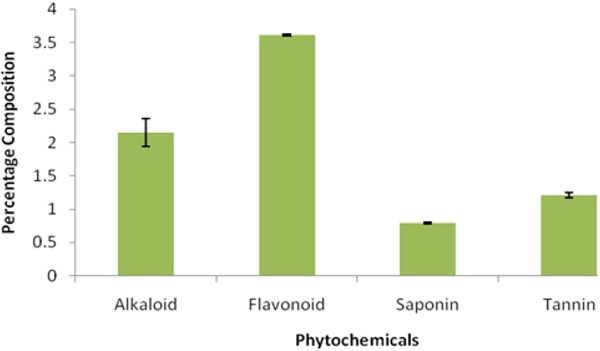
Phytochemical composition of ginger incorporated feed.

The phytochemical assay of the ginger incorporated feed contained on the average, 3.61% flavonoid, 2.15% alkaloid, 0.79% saponin and 1.23% tannin (Figure 2).

## DISCUSSION

The choice of 10% ginger being incorporated into the test feeds of the experimental rats of group 3, stemmed from the fact that ginger is merely used as a food adjunct or spice (not more than 10% of its is added into foods).

The STZ rat model of diabetes is one of the most commonly used models of human disease (24) because it mimics many of the acute and chronic complications of human diabetes and the model has the advantage of being highly reproducible. In addition, the established similarities of some of the structural, functional and biochemical abnormalities to human disease makes it an appropriate model to assess mechanisms of diabetes and evaluate potential therapies.

The destruction of the insulin secreting β-cells starts three days post STZ administration, reaching its peak at 3 to 4weeks in rats, leaving less active cells which results in a diabetic state (1; 18). This explains the development of diabetic condition in the STZ administered rats by the 3^rd^ day of STZ induction. Findings from this study indicated that incorporation of 10% ginger into the feed of the diabetic rats led to a 29.81% decrease of their hyperglycemia by the last week of the experimentation suggesting the ameliorating potentials of ginger on hyperglycemia. Our findings follow the same trend of the previous reports of Zainab *et al*. (39). However, they reported a 52% decrease in hyperglycemia which could be attributed to the duration of their experiment (6 weeks).

Urinalysis is conducted in almost all disease cases because of its enormous prognostic and diagnostic significance (8).

The excretion of large amounts of glucose in the urine (glucosuria) of the STZ administered rats is a pointer that their renal threshold of glucose was exceeded since glucosuria occurs when the filtered glucose exceeds the Tm for glucose re-absorption.

Usually, urine consists of very small (trace) amount of proteins. This is because the glomerular membrane permits only very small amount of plasma proteins (7). In 24hr urine, 1-14 mg/dl of protein may be excreted by the normal kidney (35) while values greater than 30 mg/dl may be indicative of significant proteinuria and may be a mixture of plasma albumins, globulins and proteins of the kidney itself. Diabetic nephropathy therefore occurs when proteins deposit in the glomerulus (6). In addition, the principal indicator of diabetic nephropathy is proteinuria (39). Thus, the occurrence of varying levels of proteinuria in the diabetic rats of groups 2 and 3, by the 1st week of the experiment may be indicative of glomerular complication. In addition, the low/ trace amounts of detectable proteins in the urine samples of the diabetic rats administered ginger incorporated feeds, by the last week of the experiment, suggest the ability of ginger to ameliorate glomerular complication in diabetics and this is the major significant finding in this present study.

Specific gravity (SPGR) is a urinalysis parameter that is commonly used in the evaluation of kidney function and it also aids in the diagnosis of various renal diseases. The kidneys of both humans and other mammals aid in the clearance of various water-soluble molecules, toxins, etc via excretion in urine. The concentration of the excreted molecules determines the urine’s specific gravity. Random urine may vary in specific gravity from 1.003 to 1.04 while 24 hour urine from normal patients may vary from 0.016 to 1.025 (15, 36). However, the specific gravity of rats varies from 1.022-1.05 (17). This is because the urine of rats is about twice as concentrated as that of humans.

The elevated levels of SPGR in the urine samples of the diabetic control rats and the diabetic rats administered ginger incorporated feed, by the 1^st^ week of experimentation, compared with the normal control rats, as observed in this study, could be attributed to the elevated levels of glucose in their urine (glucosuria) as well as protein in their urine (proteinuria) and this may be indicative of other substances that may have permeated the membrane of the glomerular filtrate and were dissolved in the urine. This also suggests in addition, severe renal complications for the rats of these groups. However, the reduction in the elevated urinary SPGR values of the diabetic rats administered ginger incorporated feed indicates the ability of ginger to ameliorate glomerular complication in diabetics.

Although STZ is a diabetogenic agent, intraperitoneal injections of it in experimental rats have been reported to induce kidney, pancreatic, liver, and uterine tumors in laboratory animals (37). This therefore necessitated the assay of the tissue weights of the non-diabetic, diabetic untreated and treated rats.

The increase in the liver weight in proportion to the body weights of the diabetic control rats and the diabetic rats administered ginger incorporated feed, compared with the non-diabetic rats, as observed in this study, is attributed to increased triglyceride accumulation leading to enlarged liver as a result of increased influx of fatty acids into the liver induced by hypoinsulinemia. The findings of this study are in agreement with that of previous researchers (13, 20).

Although the relative liver weights of the diabetic control and diabetic rats administered ginger incorporated feed showed a significant increase (*P*<0.05) compared with the non diabetic rats, the relative kidney weights of the diabetic rats administered ginger incorporated feeds were significantly lower than that of the diabetic control rats (*P*<0.05), indicating the kidney ameliorative potential of ginger in diabetic by maintaining or regenerating the renal cell histo-architecture.

The loss of weight and decrease in growth rates in the diabetic control rats despite their increased feed intake, is attributed to the fact that STZ-induced diabetes is characterized by severe loss in body weight and this reduction is due to loss or degeneration of structural proteins, as the structural proteins are known to be a major contributor to body weight. Again, its also indicative of the food not being transformed into weight gain or growth attainment.

The results of the TLC antioxidant screening of the ginger feed, confirms our earlier reports (10) on the antioxidant activities of the methanolic extracts of ginger.

Analysis of the chemical composition of the flour of the ginger incorporated feed indicated that it contained low quantities of moisture but significant quantities of crude fibre, proteins, lipids, carbohydrates, ash, energy values, alkaloids, flavonoids, saponins, tannins, calcium, magnesium, potassium, iron, zinc and phosphorous.

Flavonoids, alkaloids, tannins and flavonoids, as polyphenolic compounds, have been associated with hypoglycemic activity (11). The inhibition of the glycolytic activity of brush border enzymes by polyphenolic compounds seems to be one of the factors that stimulates hypoglycemic action in some medicinal plants (9). In addition, flavonoids, as antioxidants may prevent the progressive impairment of pancreatic beta cell function due to oxidative stress, thereby reducing the occurrence of diabetes. Flavonoids like myricetin, a polyhydroxylated flavonol, stimulate lipogenesis and glucose transport in the adipocytes, hence lowering blood sugar (11, 12). The alkaloid 1-ephedrine promotes the regeneration of islets of the pancreas, following destruction of the beta cells, hence restoring the secretion of insulin and thus corrects hyperglycemia (11). Tannins inhibit the activities of digestive enzymes such as trypsin and amylase. The tannin epigallo-catechin-3-gallate has been reported to exhibit anti-diabetic activity demonstrated (5).

Iron influences glucose metabolism, insulin action as well as interfering with insulin inhibition of glucose production by the liver (31).

Magnesium is a cofactor of the glycolytic enzyme hexokinase and pyruvate kinase. It also modulates glucose transport across cell membranes (28, 32). Zinc plays a key role in the regulation of insulin production by pancreatic tissues and glucose utilization by muscles and fat cells (34). Zinc also influences glyceraldehyde-3-phosphate dehydrogenase in the glycolytic pathway (23).

Dietary fibre decreases the absorption of cholesterol from the gut in addition to delaying the digestion and conversion of starch to simple sugars, an important factor in the management of diabetes. Dietary fibre also functions in the protection against cardiovascular disease, colorectal cancer and obesity (27). Thus, its plausible to assume that the presence of these compounds in ginger could play key roles in its hypoglycemic actions as well as ameliorating actions of the diabetic glomerular hypertrophy.

## CONCLUSION

The study showed that the use of ginger flours in the dietary management of diabetes mellitus could be a breakthrough in the search for plants that could prevent the development of diabetic glomerular pathology.
